# Measuring the Dynamic Nanometric Contact Radius of a Single Microdroplet on an Electrified Microinterface

**DOI:** 10.1002/anie.1113423

**Published:** 2026-03-18

**Authors:** Kathryn J. Vannoy, Jeffrey Dick, Marc Koper

**Affiliations:** ^1^ Leiden Institute of Chemistry Leiden University Leiden The Netherlands; ^2^ Department of Chemistry Elmore Family School of Electrical and Computer Engineering Purdue University West Lafayette Indiana USA

**Keywords:** contact radius, electroanalysis, microdroplet, microelectrode collisions, wetting dynamics

## Abstract

Aqueous microdroplets have received attention due to their peculiar physicochemical properties, such as their ability to drive unfavorable chemical reactions orders of magnitude more quickly than in the bulk phase. However, very few techniques can probe microdroplets, one‐at‐a‐time. Even fewer techniques can also provide real time information on the physical properties of the microdroplet reactor at the nanoscale. Such properties are highly important for rigorous mechanistic investigations. Here, we demonstrate a simple electrochemical method to quantify the nanometer contact radius that forms between a colliding droplet and an electrified surface, as a function of time. We address the limitations in the previous model used for sizing the nanometric contact area and offer a new quantitative framework. These new analyses give access to nanoscale wetting dynamics of individual microdroplets on an electrified micro‐interface. We demonstrate control over the microdroplet wetting dynamics using electrostatics. Finally, we use this platform to drive reactions within individual adsorbed microdroplets. We track the oxygen reduction reaction in real time at the well characterized microdroplet|microelectrode contact, extracting the actively partition‐controlled oxygen concentration in single microdroplets. These results have high sensitivity, allowing us to decipher both physical properties of droplets far below the diffraction limit of light and measure reactions at nanoscale, multiphase surfaces.

## Graphical abstract

Electrochemical measurements as single aqueous microdroplets collide with electrode interfaces reveal nanoscale wetting dynamics. By tuning electrostatic repulsion, we control contact formation and probe surface interactions well beyond the optical diffraction limit. Once stabilized, the droplets act as tiny electrochemical cells, allowing for direct monitoring of the oxygen content within individual microdroplets.

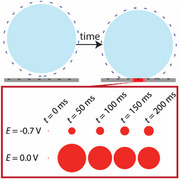

## 1 Introduction

In recent years, several groups have demonstrated the remarkable chemistry of microdroplets [1]. Cooks and coworkers have used electrospray to create droplets, where they have shown reaction acceleration and the microdroplet's ability to act as a scaffold to make peptides from free amino acids [2]. Zare and colleagues have studied other important reactions in electrosprayed and nebulized droplets, detailing a microdroplet's extraordinary ability to drive reduction reactions [3]. Recently, Lin et al. implicated contact electrification in creating electric fields to drive unfavorable reactions [4, 5]. Sharpless and Marcus reported “On Water Catalysis,” [6, 7] a phenomenon where organic reactions were accelerated at the boundary of organic solvents and water droplets, and the observed acceleration factor was hypothesized to be dictated by the interfacial area.

Much of the interest has focused on the chemistry occurring in or around the microdroplet, where the physical parameters of microdroplets (i.e., microdroplet geometry during the measurement) are relatively unresolved. Because the mechanisms suggest action at the interface [1, 7, 8], geometric properties are important for robust quantification and detailed mechanistic investigations. Griffiths and coworkers demonstrated the importance of microdroplet size considering molecular adsorption to the liquid|liquid interface of microfluidic droplets to drive reaction acceleration [9]. Huck accurately quantified an “on water” catalysis effect (organic reaction acceleration at the water phase boundary [6, 7]), by rigorously controlling the size and available surface area of microdroplets [10]. Lemay demonstrated a nanocapacitor array platform that electrochemically imaged stochastically‐colliding microdroplets based on the differences of the dielectric constant of liquid phases, mapping the multiphase environment [11].

Due to its sensitivity and quantitative power, electrochemistry has become a compelling tool to study single entities on the micro‐ and nanoscale [12, 13]. Such studies have revealed interesting insights into biological systems, using simple constructions as model systems. Vannoy et al. previously demonstrated that apparent kinetic constants increased by up to two orders of magnitude for enzymes contained in nanodroplets compared to enzymes in bulk systems [14]. Other groups have used electrochemistry to study the vesicle release of neurotransmitters, quantifying the physics and chemistry of single‐vesicle exocytosis with an unprecedented level of detail [15, 16, 17]. Beyond biochemical intrigue, uncovering the fundamental interactions between surfaces has also lead to advances in the formation of materials [18, 19, 20], where gaining better control over microdroplet surface wetting [21, 22, 23, 24] will allow for the optimization of many devices [25, 26, 27, 28].

In this study, we suggest a new physico‐mathematical model to describe the wetting profile of a microdroplet on a microelectrode surface. We demonstrate control of the wetting dynamics by modulating the electrostatic interaction between the microdroplet surface and the wetting surface. Finally, we drive the oxygen reduction reaction inside the adsorbed phase to demonstrate the quantitative power enabled by rigorous extraction of the contact area.

## 2 Results and Discussion

**FIGURE 1:**
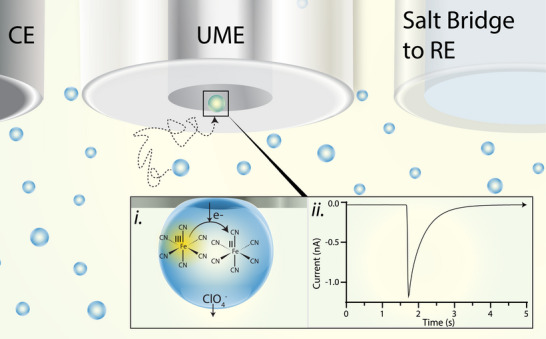
Scheme illustrating a typical collision experiment. A three‐electrode system is used with a platinum wire counter electrode, a platinum microelectrode, and a salt bridge to a Ag/AgCl reference electrode. These electrodes are submerged in an emulsion consisting of aqueous microdroplets containing 50 mM hexacyanoferrate(III) and 100 mM sodium perchlorate suspended in a dichloroethane continuous phase. (i) When a microdroplet collides with the microelectrode surface biased at a sufficient reducing potential, the hexacyanoferrate(III) is reduced to hexacyanoferrate(II) and perchlorate ions leave the droplet. (ii) In the amperometric *i*–*t* trace, the collision appears as a “blip” of current, returning to a baseline when the hexacyanoferrate(III) is completely electrolyzed. The amperogram is plotted in IUPAC convention, such that cathodic current is negative.

Electrochemical experiments are sensitive enough to measure sub‐nanometer contact radii. Picoampere current is routinely observable [29] on micro‐ and nanoelectrodes [30, 31] and electrochemistry has been used to measure down to a single platinum atom [32]. As shown in Figure 1, if a microelectrode is submerged in an emulsion, a micro‐ or nanodroplet can collide and irreversibly adsorb to the microelectrode surface, forming a nanoscale contact area where the microdroplet contents can be electro‐analyzed (Figure 1i). The stochasticity of the collision isolates the reactor in time and space. Due to the small volume of the microdroplet, the electroactive contents can be consumed in seconds (or less).

Here a single microdroplet (radius ∼1 µm) adsorbs to a biased platinum microelectrode (inlaid disk electrode of radius 5–12.5 µm). The heterogeneous reduction of hexacyanoferrate(III) occurs and transients of cathodic current are observed in the amperometric response (Figure 1ii). We include perchlorate in the aqueous phase to transfer from the aqueous droplet into the dichloroethane phase, maintaining charge neutrality. The ion transfer is not expected to have an influence the measured current (discussed in the Supporting Information and Figure S1).

**FIGURE 2:**
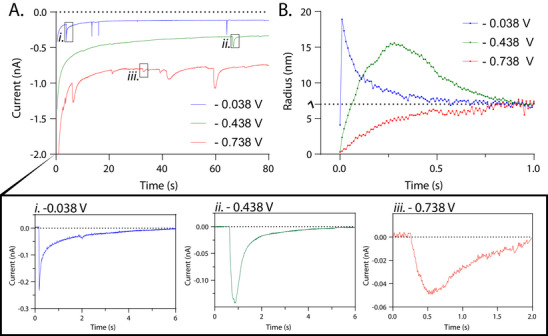
Overlay of representative amperograms collected in an Argon saturated glovebox. The working electrode was biased at −0.738 V versus Ag/AgCl (red), −0.438 V versus Ag/AgCl (green), and −0.038 V versus Ag/AgCl (blue). Selected collisions for each amperogram are indicated by overlaid black boxes and expanded in background subtracted panels (i–iii) below. (i) Example transient from the amperogram collected with the working electrode biased at (i.) −0.038 V versus Ag/AgCl (blue), (ii.) −0.438 V versus Ag/AgCl (green), and (iii.) −0.738 V versus Ag/AgCl (red). For all panels in this figure, a three‐electrode setup was used with a platinum microelectrode (25 µm diameter) as the working electrode, a Ag/AgCl reference electrode (sat'd KCl converted to 1 M KCl potentials) and a platinum wire counter electrode. The sampling rate was 100 Hz.

Throughout this study, we are applying overpotentials that are sufficient to reduce hexacyanoferrate(III) at mass‐transfer limited rates (≤ 0 V versus Ag/AgCl). Overpotentials beyond −0.75 V were not investigated to avoid high currents from water reduction in the droplet (Figure S1). Figure 2 shows the amperometric traces collected over this range of overpotentials.

Figure 2A shows an overlay of representative amperograms, where representative collision events are indicated by the boxes and expanded in panels i–iii. The transients collected with the −0.04 V‐biased microelectrode jump to a cathodic peak current and then follow a nearly exponential decay function back to the original baseline. This shape is similar to other attoliter–femtoliter electrolysis reactions previously reported in the literature [33, 34, 35, 36, 37, 38]. However, transients observed while applying a relatively high overpotential, −0.74 V, are characterized differently. These transients do not have a single point peak, but rather reach a relatively steady cathodic current before the current decays back to a baseline. Transients collected at an intermediate potential show intermediate characteristics. Thus, we observe a clear qualitative trend for the voltage dependence of microdroplet wetting: increased rounding of the current‐time transient with increasingly negative potentials.

One can extract quantitative information by integrating the current transients. The total charge (*Q*) is related to the number of moles through Faraday's Law:

(1) $$\int i\left(t\right)dt=Q=nFm=nFCV$$

where *n* is a stoichiometric constant, *F* is Faraday's constant, *m* is the total number of moles of hexacyanoferrate(III), *C* is the concentration of hexacyanoferrate(III), and *V* is the volume of the microdroplet. Assuming a spherical geometry, we can calculate the radius (*R*_d_) of the microdroplet by the total charge passed:

(2) $$\;R_{d}=\sqrt[{3}]{\frac{3Q}{4\pi nFC}}$$

As shown in Table S1, though the current transient shape changes based on the applied potential, the total charge remains similar. This is expected since microdroplet size should be independent of electrode charge.

Bulk electrolysis equations are canonically used to calculate the contact radius by fitting the transient decay [38, 39, 40]. The rate of consumption is controlled either by heterogenous kinetics or mass transport, depending on the applied potential. One can extract heterogenous rate constants for reactions in low dielectric media by applying low overpotentials [39]; otherwise, a potential just beyond the mass transfer limitation is selected such that there is only one adjustable parameter in fitting, namely, the effective electrode area term (i.e., the contact area) [38]. The bulk electrolysis equation for microdroplet consumption is described in detail in the Supporting Information and Figure S2.

One can readily see that all the current transients in Figure 2 would not be fit by a single exponential function. Similar deviations are also given in the literature, where data from first ∼200 ms of the current transient are ignored such that bulk electrolysis equations can be used [38]. Because these deviations appear at the early timepoints (i.e., current transient rounding), and seem to be potential‐dependent (Figure 2), we suggest that wetting dynamics may be responsible and measurable in these experiments. We hypothesize that the more negative bias increases repulsion effects between the microelectrode surface and the suspended microdroplets, which carry a negative surface charge. The negative surface charge of the microdroplets was confirmed with zeta potential measurements (−73.5 ± 12.5 mV, *N* = 3). Though we do not precisely know *E*_pzc_ under these experimental conditions, more negative potentials charge the electrode surface more negatively.

**FIGURE 3:**
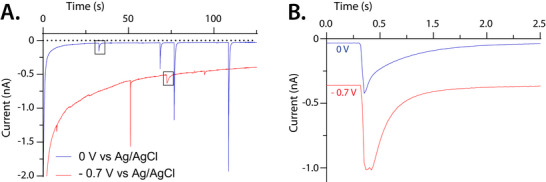
(A) Overlay of representative amperograms collected with the working electrode biased at −0.7 V versus Ag/AgCl (red) and 0 V versus Ag/AgCl (blue). The black boxes enclose the individual current transients overlaid in Panel B. (B) Example transients from the amperograms collected with the working electrode biased at −0.7 V versus Ag/AgCl (red) and 0 V versus Ag/AgCl (blue). The transients were time normalized to show duration. For all panels in this figure, a three‐electrode setup was used with a platinum tip microelectrode (10 µm diameter) as the working electrode, a Ag/AgCl reference electrode and a platinum wire counter electrode. The emulsion solution consisted of 35 µL 50 mM hexacyanoferrate(III) and 100 mM sodium perchlorate in 5 mL 0.5 M tetrabutylammonium perchlorate in dichloroethane. The sampling rate was 60 Hz.

While the initial experiments were performed in a glovebox, to avoid contributions from oxygen, we found that collisions under ambient atmosphere give a similar potential dependency (Figure 3). This observation falls in in line with wetting dynamics, which we would not expect to be significantly impacted by background current contributions (i.e., the oxygen reduction reaction occurring after the microdroplet has made contact, discussed in detail below). This also simplifies experimental procedures; thus, the data in the remainder of the figures was collected under ambient conditions.

Figure 3A shows representative amperograms collected at high and low overpotentials within the mass transport limited window of hexacyanoferrate(III) reduction. Figure 3B compares the collision response of similarly sized microdroplets (*R*_d_ ∼1.9 µm). Figure S3 shows our attempt to fit the transients shown in Figure 3B with bulk electrolysis equations to extract the contact radius, as is done canonically in the literature. For the transient collected at 0 V, the decay across timepoints 0–0.5 s is too sharp for the remaining exponential decay (Figure S3A). The transient collected at −0.7 V is rounded until 0.22 s after the first point, where it then follows an exponential decay (Figure S3B). Importantly, in order to fit bulk electrolysis equations, the current must decay in a predictable shape from the first timepoint (*i*_0_), precluding the possibility of a time‐dependent variable (i.e., contact radius, *r*_c_). Practically, this means that the contact radius is considered constant and microdroplet wetting is assumed to be instantaneous.

We propose analyses that do not fix *r*_c_ but instead calculate the contact radius at each timepoint to quantify a “wetting” regime. The limiting current (*i*_lim_) to an inlaid disk micro‐ or nanoelectrode is given by the following expression:

(3) $$i_{\lim}=4nFDCr$$

When analyzing each timepoint individually, this becomes:

(4) $$r_{c}\left(t\right)=\frac{i_{\lim}\left(t\right)}{4\mathrm{nFDC}\left(t\right)}$$

where *r*_c_(*t*) is the contact radius, *i*_lim_(*t*) is the limiting current, and *C*(*t*) is the concentration, all calculated at timepoint *t*. This value can be calculated for each measured timepoint on the transient. The full derivation of Equation (4) and further discussion is given in the Supporting Information and Figures S4–S5).

**FIGURE 4:**
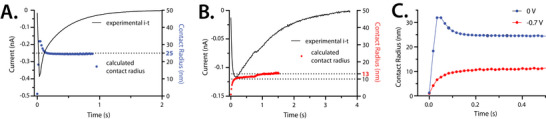
(A) Analysis of a collision collected at 0 V versus Ag/AgCl. Plot of Equation (4) (right *y*‐axis) overlaid with the experimental data (left *y*‐axis). Values for *i_t_* were taken as each sampling point of the experimental data (black trace) and *Q* corresponded to 26.7 fL through Equation (1). The dotted line corresponds to 25 nm on the right *y*‐axis. (B) Analysis of a collision collected at 0 V versus Ag/AgCl. Plot of Equation (4) (right *y*‐axis) overlaid with the experimental data (left *y*‐axis). Values for *i_t_* were taken as each sampling point of the experimental data (black trace) and *V* was 30.0 fL. The dotted lines correspond to 10 and 13 nm on the right *y*‐axis. (C) Overlay of the contact radii versus time over the first 0.5 s of the collision events shown in Panels A and B. The blue dots correspond to the collision in Panel A, where the microelectrode was biased at 0 V versus Ag/AgCl (1 M KCl). The red dots correspond to the collision in Panel B, where the microelectrode was biased at −0.7 V versus Ag/AgCl.

Figure 4A,B show an overlay of the background subtracted current transients versus time with the contact radius calculated at each collected timepoint by Equation (4). We compare microdroplets of a similar size, both ∼1.9 µm in radius. For high confidence in the quantification, we only plot the contact radii calculated from currents that were 10X the noise. In these experiments a typical peak‐to‐peak noise was ∼3.5 pA. Calculating the contact radius with currents below 3X noise (∼10 pA) showed noticeable error (Figure S6). The analyses in Figure 4A,B explain the deviations from the electrolysis equation as time‐dependent changes in the contact area between the microdroplet and microelectrode during wetting. Additionally, Figure 4C shows that the wetting function depends on the electrode potential, a parameter that is often neglected beyond its influence on the electron transfer reaction rate.

For collision events on a more negative surface (−0.7 V), the negatively charged microdroplets gradually wet to reach the stable contact radius (more examples, Figure S7). This contrasts with collision events on a mildly biased surface (0 V), where microdroplets quickly overshoot the stable contact radius, and then rearrange to a stable contact (more examples, Figure S8). After about 200 ms, the contact radii become relatively stable at both potentials. On the 0 V‐biased microelectrode surface, the stable contact radii scale linearly with droplet volume (∼10–100 fL), in line with previous reports [41]. We observed less stability in the contact radius over time for microdroplets on the −0.7 V‐biased surface and no clear trend with microdroplet volume, which may suggest that the microdroplets do not pin strongly. The contact radii calculated (10–30 nm for a 30 fL microdroplet) fall in line with previous reports that use these analyses for similar microdroplet systems [38, 40] and electron microscopy data that validates nanometer contacts for micrometer droplets [42].

The rounded transients are always observed when applying −0.7 V (*N* = 25), and the single‐point peak is observed 80% of the time (*N* = 25) for the 0 V condition. Six collision events were quantitatively analyzed from each potential regime and 100% of these agreed with the described wetting response (Figures S7 and S8 show replicates to those in the main text). Quantified collision events prioritized early events (the first or second blip observed) and those with the clearest baselines, to minimize the inclusion of collisions with droplet–droplet interactions. Experiments at intermediate potentials (−0.44 and −0.54 V versus Ag/AgCl) show a qualitative wetting profile between these observations, where there is a broad overshoot of the contact radius (Figure S9–S10). Figure S10 also confirms that a similar voltage‐dependent shape relationship trend is observed for the collisions collected under Argon atmosphere. Figure S11 shows the qualitative relationship for the collisions of smaller (radii ∼100 nm) droplets.

Previous reports have shown that the hydrophilicity of the microelectrode [43] and insulating glass surface [44] influences the collision dynamics, but the role of electrode surface charge has not been examined. We suggest that the highly negative surface (−0.7 V) leads to an electrostatic repulsion between the negatively charged microdroplets and the electrode surface, slowing the velocity of the microdroplet near the like‐charged surface [45]. Thus, the overshoot seen in seen in the mildly‐biased case is not observed for collisions on highly negative surfaces (−0.7 V). We further tested the electrostatic theory by measuring collision responses of more positively charged droplets. We found that we were able to recover the sharper decay shape and wetting overshoot (Figure S12–S13) at very negative potentials (−0.738 V versus Ag/AgCl). As the current is mass transport limited in all of the conditions outlined, there can be no electrochemical reason for the observed potential dependence.

Interestingly, we note that there is near equivalence between Equation (4), and the equations that describe vesicle release through pore opening [46, 47]. While the microdroplet and vesicle systems have unique differences, both assume that transport within the microsphere is rate‐limiting. The slight difference in flux patterns through a disk opening along the circumference and to a planar disk electrode is very minor. Importantly, transport through the pore channel (through the vesicle membrane) can usually be neglected due to the slower diffusion rates inside the vesicle compared to the extracellular solution [46, 47]. Thus, the observed current responses, and subsequent time‐dependent radii conclusions, are analogous between systems.

**FIGURE 5:**
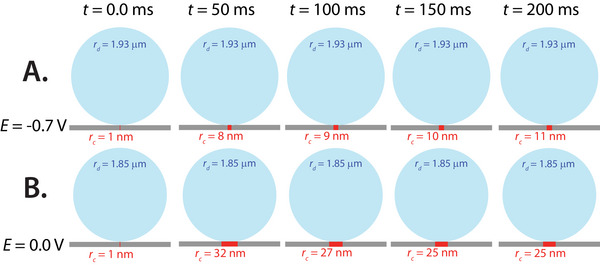
2D scheme of the electrochemically observed wetting presented in Figure 4. (A) The top row corresponds to a collision event occurring on a platinum microelectrode biased at −0.7 V versus Ag/AgCl (1 M KCl). (B) The bottom row corresponds to a collision event occurring on a platinum microelectrode biased at 0.0 V versus Ag/AgCl (1 M KCl). The columns correspond to ∼50 ms intervals after the first point on the collision transient (0.0 ms). The contact radii are illustrated by red boxes on the grey microelectrodes, where the contact radii are relatively to scale with each other but the microdroplets (illustrated in blue) are reduced by an order of magnitude relative to the contact radii for ease of viewing.

The method presented in this study gives a dynamic picture of a microdroplet wetting on an electrified micro‐interface, so in Figure 5, we present a 2D timelapse schematic series illustrating a microdroplet collision event occurring at a microelectrode biased at 0 V (Figure 5A) and −0.7 V (Figure 5B). We show intervals of 50 ms for concise representation of the overall wetting dynamics observed, but we note that the time interval can be as low as the sample rate. The sampling rate of 60–100 Hz was chosen as it is comparable to typical sample rates for microdroplet collision experiments in the literature. We find that wetting does significantly influence the collision response under such conditions. Future directions are aimed at fine‐tuning these measurements to reach kHz bandwidth to uncover further details about dynamic wetting behavior.

Finally, with knowledge of how individual microdroplets sit on the surface, reaction quantitation becomes possible. One can see in Figure 3 that collision responses collected at −0.7 V, under ambient conditions, have a slightly elevated baseline after the hexacyanoferrate(III) reduction. Steady‐state oxygen reduction in the aqueous microdroplet sets this baseline, as is clearly evidenced by the return to the original baseline under anaerobic conditions (Figure S14–S15). Importantly, this reaction can be viewed as background current in the hexacyanoferrate(III) reduction as it is steady‐state and an order of magnitude lower than the peak currents. Thus, in the analyses above, the baseline for quantification of the charge from hexacyanoferrate(III) reduction was drawn from the limiting current observed after the current transient (Figure S16).

Because of the fast mixing within the microdroplets, the elevated baseline (shown in Figure S16 as a red line at *x* = 9.45 nA) reports on the aqueous oxygen content. The oxygen is not consumed as oxygen dissolved in the dichloroethane partitions into the microdroplet. The −0.7 V versus Ag/AgCl bias is sufficient to drive aqueous ORR at mass transfer limited rates (before the pH changes significantly enough to slow the reaction). Thus, one can estimate the oxygen content of single microdroplets using Equation (3), where *D* is 2 x 10^−9^ m^2^∙s^−1^ [48], *r* is the stable contact radius calculated by Equation (4), and *n* is a value between 1 and 4 [49, 50, 51]. These calculations gave a range of 0.3–1.2 mM (Figure S17), for *n* = 4 and *n* = 1, respectively. If we assume 4 electrons, the extracted value is very close to what is expected from Henry's law. The agreement further suggests the validity of the calculated contact radii and acts as proof‐of‐concept for using the well characterized microdroplets as continuous femtoliter reactors (similar in size to cells and atmospheric aerosols) for interesting reactions. While oxygen reduction can be quite sluggish, we apply a sufficiently high overpotential for the reaction to be mass transport limited, thus we can safely assume that the electron transfer rate is not kinetically limited. Additionally, partition kinetics of oxygen across the dichloroethane| aqueous boundary has previously been demonstrated to be very fast [52], which is in agreement with our finding that mass transfer‐limited equations give the expected microdroplet oxygen concentration (thus, no apparent kinetic limitations). However, we note that this work does suggest the possibility to study single micro‐ and nanodroplet partition kinetics [53], an important topic in fundamental atmospheric [54] chemistry and biochemistry [55]. The real time measurement of hindered partitioning of gases [56] is a clear future direction of this work, especially in the contexts of nanomedicine [57].

## 3 Conclusion

When a microdroplet irreversibly adsorbs to a microelectrode, a very small contact area forms. We provide an equation that allows for the elucidation of the entire wetting process for single aqueous microdroplets as they actively wet a microelectrode surface. We demonstrate control over the wetting dynamics by increasing the repulsion between the microelectrode surface and the microdroplets. When both surfaces are significantly negative, the microdroplet wetting becomes gradual, avoiding contact overshoots. Finally, we show that we can use the adsorbed microdroplets as tiny electrochemical cells for aqueous oxygen reduction reaction. The elucidated contact radii are used to estimate the concentration of dissolved oxygen within each microdroplet, which was found to be ∼0.3 mM, agreeing well with theoretical values. This method is unmatched in its sensitivity to quantify the dynamic microdroplet geometries well below the diffraction limit of visible light.

## 4 Materials and Methods

### 4.1 Chemical and Materials

Potassium chloride (KCl, >99%), potassium hexacyanoferrate(III) trihydrate (ferricyanide, ≥99%), and agarose were obtained from Fisher (Fair Lawn, NJ). Tetrabutylammonium perchlorate (TBAP, ≥99.0%), sodium perchlorate (NaClO_4_, ≥98.0%), and dichloroethane (DCE, anhydrous, >99.8%) were obtained from Sigma‐Aldrich (St. Louis, MO). Aqueous solutions were prepared with deionized water (18.20 MΩ·cm) from a Barnstead GenPure Milli‐Q water purification system, which was purchased from Thermo Scientific (Waltham, MA). Platinum SECM microelectrodes (*r* = 5 µm) and silver/silver chloride (Ag/AgCl, 1 M KCl) reference electrodes were purchased from CH Instruments (CHI, Austin, TX). A platinum wire (*d* = 1 mm) from GoodFellow (Pittsburg, PA) was used as the counter electrode. A salt bridge was constructed from a glass tube filled with 3% w/w agarose/1 M KCl. A horn sonicator (QSONICA Q500) was used to create all emulsions (500 W, 20 kHz). The tip was thoroughly cleaned with ethanol before and after each emulsion preparation.

For experiments in Figure 1; Figures S1 and S7–S12 potassium hexacyanoferrate(III) trihydrate (ferricyanide, ≥99%) and sodium perchlorate hexahydrate (≥99.9%) were obtained from Sigma Aldrich. Miniature leakless silver/silver chloride (Ag/AgCl, sat'd KCl) reference electrodes were purchased from eDAQ and used without a salt bridge. A platinum wire (*d* = 1 mm) was used as the counter electrode and a platinum microelectrode (*r* = 5 µm or *r* = 12.5 µm) obtained from CH Instruments and used as the working electrode.

Glovebox experiments were performed in a mBRAUN Labmaster sp purged with Argon gas. The sonicator, faraday cage, and potentiostat were all operated inside the glovebox. The prepared electrolyte solutions were introduced to the glove box in 15 mL volumes one day‐one week before measurements, kept loosely capped to minimize solvent evaporation.

### 4.2 Collision Experiments

Most electrochemical experiments were performed with a CH Instruments 6284e potentiostat and a three‐electrode setup. Amperometric traces were obtained in emulsion solutions using a three‐electrode configuration where a platinum microelectrode, a platinum wire, and a Ag/AgCl (1 M KCl, salt bridge) electrode were used as the working, counter, and reference electrode, respectively. The working and counter electrodes were placed directly in the emulsion solution and the reference electrode was connected to the reference solution by a salt bridge. To create the emulsion solution, ∼35 µL of aqueous solution was emulsified into a 5 mL DCE solution using a pulse method at 40% amplitude (5 s on, 5 s off, 60 s). A potential of −0.7 V versus Ag/AgCl (1 M KCl, salt bridge) was applied to the microelectrode for 100 s, sampling at 60–100 Hz.

For experiments in Figure 1; Figures S1 and S7–S12: performed as above with a Biologic SP‐300 Potentiostat with an ultralow current module. A miniature leakless Ag/AgCl (saturated KCl) reference electrode was used, and the reported potentials were converted to the 1 M Ag/AgCl reference by accounting for a 38‐mV negative shift.

### 4.3 Zeta Potential Measurements

Zeta potential measurements in Table S1 were performed using a Malvern Zetasizer Pro instrument and a ZEN1002 dip cell kit from Malvern Panalytical (Worcester, UK).

## Conflicts of Interest

The authors declare no conflicts of interest.

## Supporting information




**Supporting File 1**: anie71637‐sup‐0001‐SuppMat.docx.

## Data Availability

The data that support the findings of this study are available from the corresponding author upon reasonable request.
