# Supplementary material for: Measuring the Dynamic Nanometric Contact Radius of a Single Microdroplet on an Electrified Microinterface
Source: Angew Chem Int Ed Engl. 2026 Mar 18;65(18):e1113423. doi: 10.1002/anie.1113423 (PMC13110780; doi:10.1002/anie.1113423)
Supplement: Supplementary file 1 — Supporting File 1: anie71637‐sup‐0001‐SuppMat.docx. [file ANIE-65-e1113423-s001.docx]

**Measuring the Dynamic Nanometric Contact Radius of a Single Microdroplet on an Electrified Microinterface**

Kathryn J. Vannoy^a*^, Jeffrey Dick^b*^, and Marc Koper^a*^

^a^Leiden Institute of Chemistry, Leiden University, PO Box 9502, 2300 RA Leiden, The Netherlands

^b^Department of Chemistry, Purdue University, West Lafayette, Indiana 47906, United States

Elmore Family School of Electrical and Computer Engineering, Purdue University, West Lafayette, Indiana 47906, United States

Table of Contents

Mass Transport Limits and Ion Transfer Discussion

Figure S1S2-3

Table of Microdroplet Sizes

Table S1S4

Bulk Electrolysis Computations

Figure S2-3S5-8

Equation 4 Validation

Figure S4-5S9-12

Contact Radii as a Function of Time (Full and Capped)

Figure S6S13

Contact radius *versus* time for Replicate Collisions (-0.7 V)

**Figure S7S14**

Contact radius *versus* time for Replicate Collisions (0 V)

Figure S8S15

Contact radius *versus* time for Representative Collision (-0.538 V)

Figure S9S16

Overpotential-dependence Under Anaerobic Conditions

Figure S10S17

Bias-Dependent Nanodroplet Collision Shapes

Figure S11S18

More Positively-charged Microdroplet Collisions

Figure S12-13S19-20

Glovebox Measurements (Anerobic)

Figure S14-15S21-22

Transient Showing the Limiting Current from Aqueous Oxygen Reduction

Figure S16S23

Calculations of Aqueous Dioxygen Concentration in Individual Microdroplets

Figure S17S24

References

S25

# Cyclic Voltammetry in the Aqueous Phase and Ion Transfer Between the Aqueous Phase and Continuous Dichloroethane Phase

Aqueous ions: K^+^, Na^+^, ClO_4_^-^, Fe(CN)_6_^3-/4-^

Organic ions: TBA^+^, ClO_4_^-^

Perchlorate (ClO_4_^-^) has the lowest Gibbs free energy of ion transfer (17.2 kJ/mol)^1, 2^, suggesting it is the most favorable to transfer. A common ion (i.e., perchlorate) between liquid phases is expected to set a liquid junction potential. **Figure S1A** shows a simple biphasic experiment where the working electrode is in the aqueous phase and the counter and reference electrodes are in the organic phase, thus seperated by the aqueous|DCE interface. **Figure S1B** demonstrates that the conditions for mass-transfer limited rates are unaffected despite the polarization aqueous|DCE interface. We overlay cyclic voltammograms to compare two aqueous conditions: [ClO_4_^-^]_microdroplet_ = 100 mM and [ClO_4_^-^]_microdroplet_ = 50 mM. A small shift (~30 mV) is observed in the *E_1/2_* of the Fe(CN)_6_^3-/4-^ couple that we attribute to a change in the liquid|liquid polarization. Assuming that 1 mole ClO_4_^-^ transfers from water to DCE for every 1 mole Fe(CN)_6_^3-^ reduced, **Figure S1** confirms that the current remains mass transfer-limited over the duration of the collision experiment (50 mM Fe(CN)_6_^3-^) and illustrates the upper limit of ~ -0.75 V for avoiding proton/water reduction. However, we note that we do not know the contribution of TBA^+^ on the ion transfer in these experiments, but find that the inclusion of NaClO_4_ in the aqueous phase avoids the loss of hexacyanoferrate(III) to the DCE phase.^4^


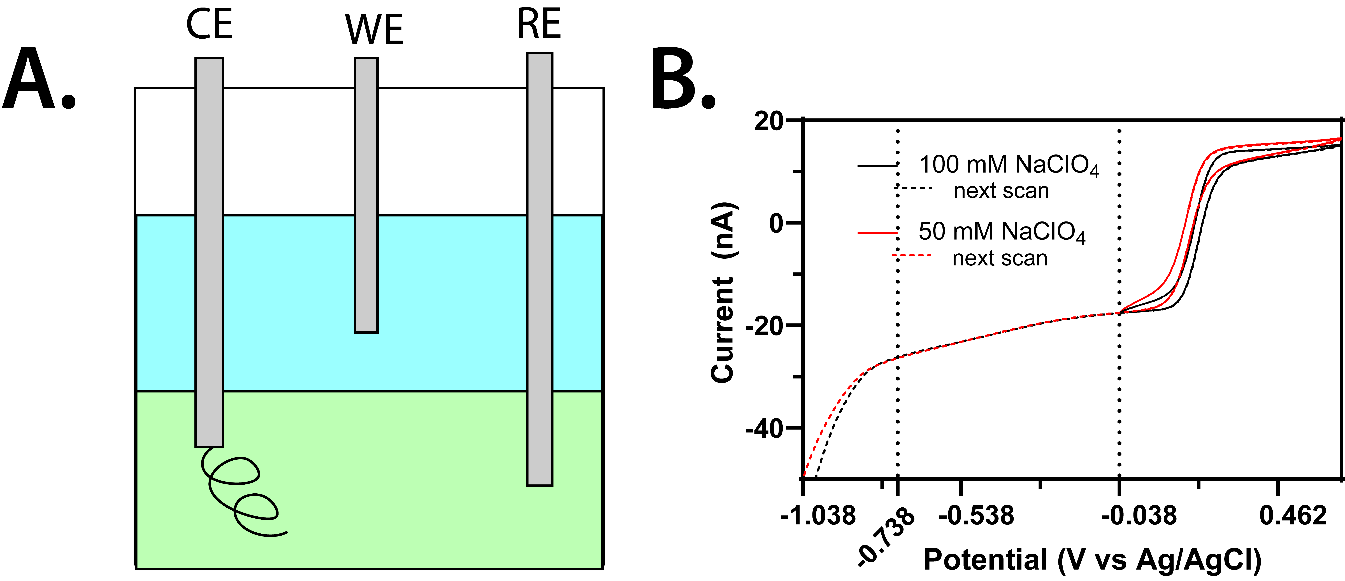
**Figure S1. (A)** Biphasic experimental setup. The aquous phase is represented in blue and consited of 5 mM potassium hecancyanoferrate(II and III), and either 100 mM sodium perchlorate or 50 mM sodium perchlorate. The DCE phase is represented in green and consisited of 500 mM tetrabutyleammonium perchlorate. The working electrode (Pt UME, *d* = 25 µm) was in the aqueous phase and the reference (leakless Ag/AgCl, sat’d KCl adjusted to the 1 M KCl potetnial scale) and counter (Pt wire electrode were in the DCE phase. **(B)** Cyclic voltammograms collected in the conditions described in panel A, where the black trace is the 100 mM sodium perchlorate condition and the red is the 50 mM sodium perchlorate condition. The correspondingly colored dashed line is the next scan to show the current at more negative potentials. Potentials at 0 and -0.7 V are are highlighted to show that the current is unaffected by the liquid junction potential. The electrochemical cell was open to air and the scan rate was 50 mV/s and the voltammograms were plotted in IUPAC convention.

# Table S1. Charge Passed During Each Collision Event

| **Bias (V *versus* Ag/AgCl)** | **Average Charge Passed per Collision Event (pC)** | **Standard Deviation on the Charge Passed (pC)** | **Number of Collisions Sample** |
| --- | --- | --- | --- |
| -0.7 | 116 | 60 | 5 |
| 0 | 249 | 238 | 5 |

The microdroplets are sized electrochemically by relating the charge passed during the current transient and the concentration of the consumed species to the microdroplet volume (Equations 1-2, main text). **Table S1** shows the size distributions measured where the microelectrode was biased at -0.7 V and 0 V. A t-test suggests that the size of the collected microdroplets is not significantly influenced (P > 0.05) by the microelectrode bias. Though the microdroplets do carry a surface charge, effects from long range electrophoretic migration^3^ is not expected under these microdroplet charge/size and continuous phase electrolyte conditions.

# The Bulk Electrolysis Equation and the Influence of the Microdroplet Contact Radius/Microdroplet Radius on the Transient Decay

The behavior of the current as a function of time (*i*(*t*)) during the electrolysis of a microdroplet is given by the following expression:

$i\left( t \right)= i_{0}e^{-\frac{k_{m}A}{V}t}=i_{0}e^{-\frac{4Dr_{c}}{V}t}$ *(S1)*

,where *k_m_* is the mass transfer coefficient, *A* is the electrode area (or contact area), and *t* is the time. The second equality is obtained by assuming the mass transfer coefficient is that to an inlaid disk electrode: $k_{m}=\frac{4D}{\pi r_{c}}$. Electrolysis begins at *t* = 0 with a current *i_0_* and the time it takes to consume the contents *(*i.e., current reaches the baseline) depends on the size of the cell (the larger the volume, the slower the consumption) and the size of the electrode (the larger the electrode, the faster the consumption).

Theoretically, the first point on the current transient should be used as *i_0_* in Equation S1. However, in practice, data from first ~200 ms of the current transient is sometimes ignored because the current-time behavior deviates from the electrolysis decay function.^5^ Thus, the value of *i_0_* is often treated loosely to better fit experiments by: (1) Taking *i_0_* as the experimental peak current (*i_p_*), which may be several hundreds of milliseconds into the current transient, or (2) allowing *i_0_* to be an adjustable parameter, where the fit is allowed to deviate at the early timepoints to prioritize fitting the majority of the transient. There is no reason given for the first case^5, 6^ (here we will propose one), but the second is often justified with the reasonable argument that sampling rates cause the true *i_0_* value to be difficult to accurately measure. This is most valid for collision events that consume very quickly. **Figure S2** shows the general influence of the droplet volume, contact radius, and sample rate. We note that a value on the order of picometers has been reported using these equations, which is a value inconsistent with continuum modeling, highlighting the uncertainty in current models.^7^

The collision current (*i*(*t*)) reports on the consumption of hexacyanoferrate(III) in an adsorbed microdroplet, and can be fit by the bulk electrolysis equation (Equation S1). Here we modulate the contact radius and droplet radius parameters in the computation to demonstrate that the sample rate provides some uncertainty in the fitting, and that the level of uncertainty is influenced by the microdroplet geometry on the electrode. **Figure S2A** shows that current decay is very sharp when the contact radius is bigger (fixed microdroplet radius), so there is more error in assuming that the peak current (experimentally influenced by the sample rate) is *i_0_*. **Figure S2B** shows that sample rate limits the information in the current decay more significantly for small droplets compared to larger microdroplets, as a majority the charge is passed within one sample interval for microdroplets under 0.3 µm (contact radii fixed directly proportion to the microdroplet size).


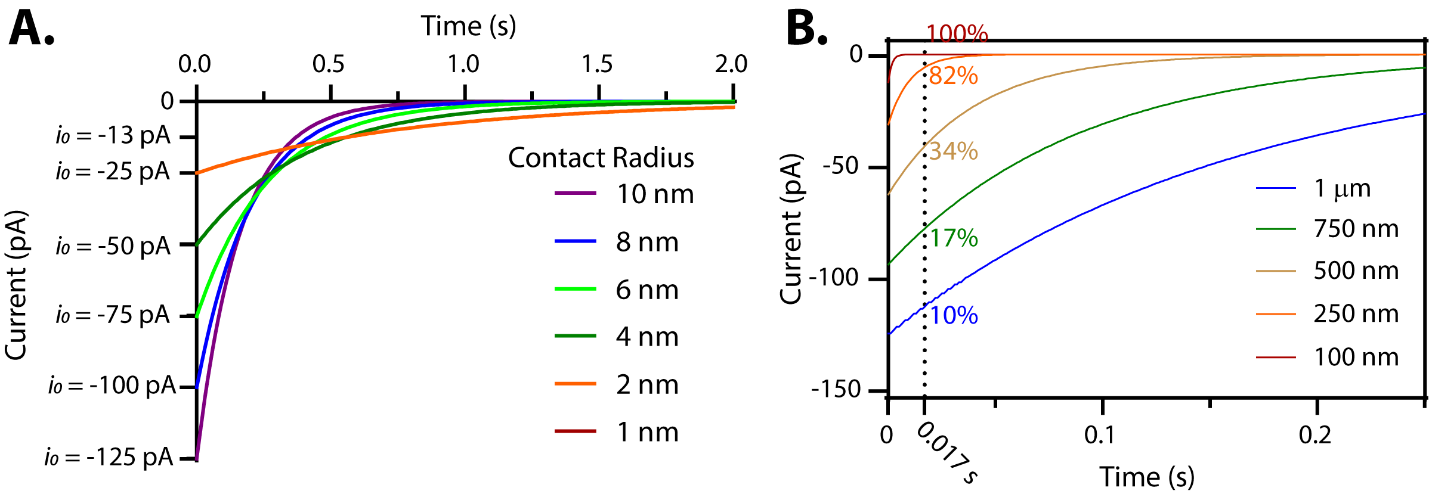


**Figure S2.** (**A**) Plot of Equation S1 where the volume is fixed at 4.2 fL (corresponding to a microdroplet of 1 um in radius) and the contact radius is changed from 1 nm (red trace) to 10 nm (purple trace). The y-axis gives the expected limiting current (*i_0_*) for each of the traces. (**B**) Plot of Equation S1 where the ratio of the droplet radius/contact radius is fixed at 100 and the droplet radius is changed from 1 µm (blue trace) to 100 nm (red trace). The dotted line is given at x = 0.017 s, as the first sampling point and the percentage of the droplet contents that is electrolysed at 0.017 s is overlaid beside the corresponding trace.

# Partial Fitting with the Bulk Electrolysis Equation

**Figure S3A** shows a transient collected at 0 V overlaid with Equation S1, which was fit to the experimental data by altering the contact radius variable. The decay across timepoints 0 – 0.5 s is too sharp for the remaining exponential decay (**Figure 3A**). Using Equation S1 for the collision collected at -0.7 V is more complicated. We take *i_0_* as the point before the current decays (0.22 s after the start of the current transient) and adjust this point at *t* = 0. While in the literature, any timepoints before the decay function are usually ignored, the transients collected here at -0.7 V pass significant charge before an exponential decay function is observed (**Figure S3B**). Thus, here we correct for this charge passed by adjusting the concentration term at *t* = 0 in the bulk electrolysis equation by partial integration (*Q_t_*) of the current transient. By assuming the microdroplets are perfectly mixed, a common assumption considering diffusion on small scales^8^, we can relate the consumed moles at each timepoint (*Q_t_*) to a new concentration in the microdroplet (*C_t_*),

*Q_t_ = nFC_t,consumed_V (S2)*

where *C_t,consumed_* is the consumed concentration at time *t*. The volume of the microdroplet is known from Equation 1, and the charge passed, and concentration consumed, are directly proportional, as shown in Equation S3.

$\frac{Q_{t}}{Q}=\frac{C_{t,consumed}}{C}$ *(S3)*

Equation S3 shows that the total charge passed at the conclusion of the current transient (*Q*) and the charge passed at timepoint *t* (*Q_t_*) is equivalent to the initial droplet concentration (*C*) and the concentration consumed at timepoint *t* (C*_t,consumed_*). Because these concentration parameters refer to the consumed concentration, the droplet concentration at time *t* is calculated by subtracting these values from the initial bulk concentration (*C* – *C_t,consumed_* = *C_t_*). For the transient shown in **Figure S3B**, *Q_t_* corresponded to the consumption of 7.6 mM, thus the concentration at *t* = 0 for the Equation S1 decay function was 42.4 mM (instead of the initial concentration, 50 mM).

**
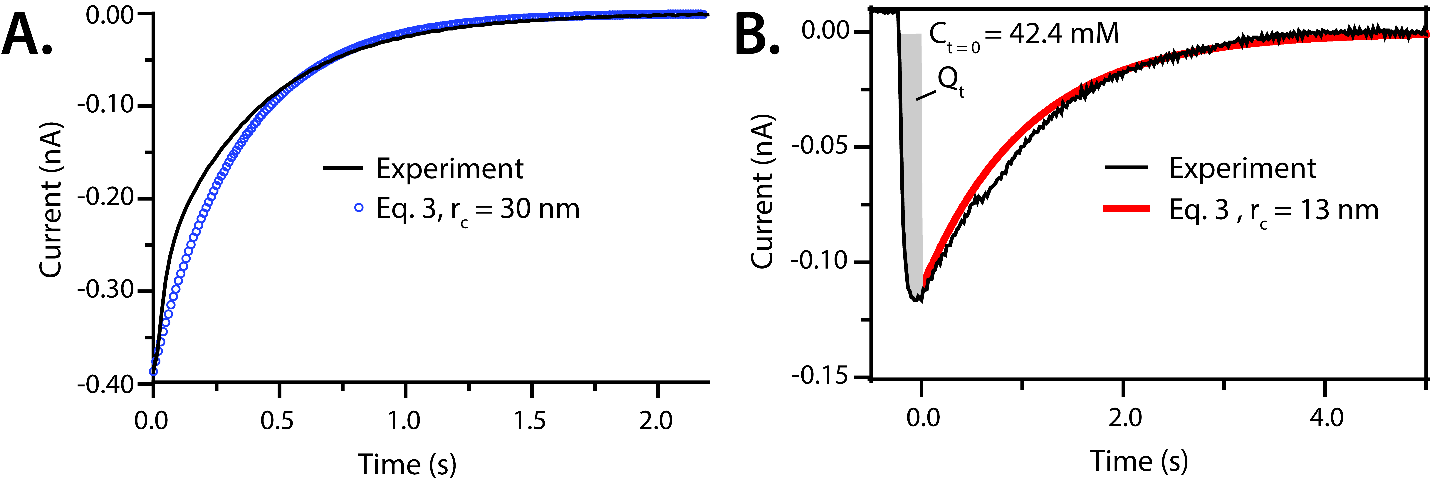
Figure S3.** **(A)** Overlay of Equation S1 with experimental data collected with a microelectrode biased at 0 V *versus* Ag/AgCl (1M KCl). The volume was fixed at 26.7 fL (as determined from Equation 1) and the contact radius (*r_c_*) was changed until the best overlay of the experimental data (black trace) was achieved (*r_c_* = 30 nm, open blue circles). The experimental data was background-subtracted such that the highest point of the experimental decay corresponded to 0.0 s and the end of the decay was 0 nA. **(B)** Overlay of Equation S1 with experimental data collected with a microelectrode biased at -0.7 V *versus* Ag/AgCl. The experimental current transient (black trace) was background-subtracted such that the exponential decay function starts at *t* = 0 and the end of the decay was 0 nA. The microdroplet volume was calculated as 30 fL (Equation 1), corresponding to a radius (*r_drop_*) of 1.93 µm by Equation 2. The grey shading illustrates integration, giving the charge passed before the current decays in a typical electrolysis function (*Q_t_*, corresponding to a concentration of 42.4 mM at *t* =0). A bulk electrolysis expression (Equation S1, solid red line) was fit to the decay, with fixed parameters *i_0_* = 0.116 nA, [hexacyanoferrate(III)] = 42.4 mM, and *V* = 30 fL, and iterated to fit *r_c_* (13 nm shown). The sampling rate for experimental data is 60 Hz.

# Equation 4 Validation

**Neglecting the Contact Angle**

Our model considers that at time 0, the microdroplet of radius *R_d_*, connects to the electrode surface forming a planar disk of radius *r_c,t=0_*. The contact angle is assumed to have a negligible impact on the measured current. **Figure S4A**, shows a nearly spherical droplet (*V* = ~ 30 fL) with the base removed to form the planar disk contact (*r_c_* = 25 nm). **Figure S4B** shows the droplet geometry is adjusted to allow for the typical hemispherical flux pattern of a planar disk nanoelectrode, while maintaining the same volume and contact radius. **Figure S4C** demonstrates there is no experimentally observable difference between the electrolysis decays in these two geometries. Thus, the equations solved for typical hemispherical diffusion are valid for these geometries, despite the small degrees of freedom lost by the contact angle We also confirm this for a microdroplet that is an order of magnitude smaller (*V* = ~3 fL, *r_c_* = 2.5 nm), confirming the validity over the range of geometries proposed in this work. We find that the contact never becomes large enough compared to the droplet to warrant consideration of the exact coefficients changing from the planar disk approximation, though we acknowledge that there are conditions where this may become important. For more information about the COMSOL model, see the attached COMSOL report.

**
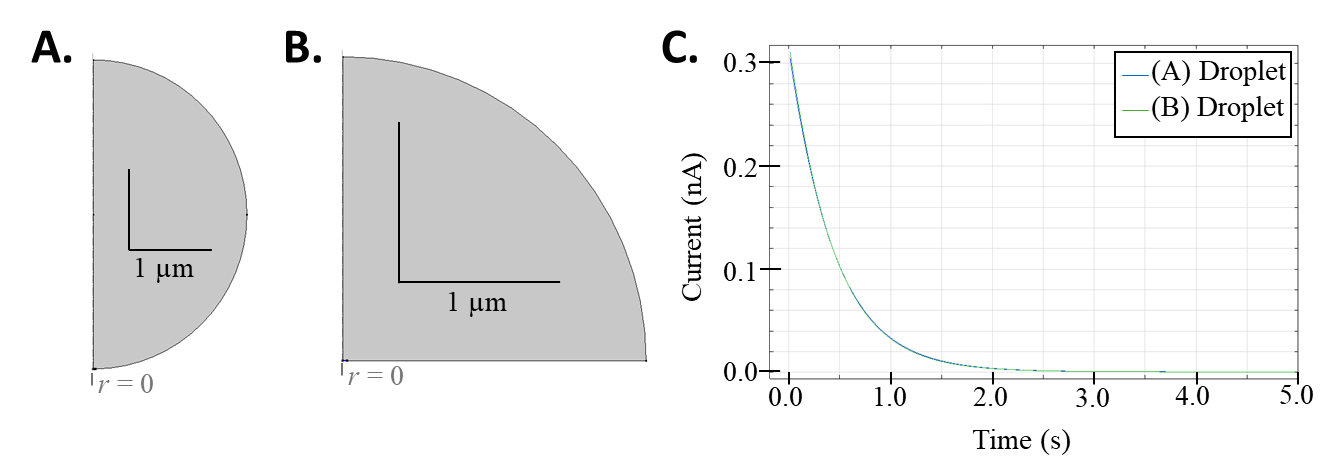
**

**Figure S4. (A)** 2D-axisymmetric geometry of a 28.7 fL (*R_d_* = 1.9 µm) droplet with axis of symmetry at *r* = 0. The contact is given by a planar cut to the base resulting in a disk with radius 25 nm, which is assigned as the electrode. **(B)** 2D-axisymmetric geometry of a 28.7 fL droplet with axis of symmetry at *r* = 0. The electrode is given by a 25 nm line segment along *y* = 0, where the remaining line segment is a no flux boundary. **(C)** Overlay of the current-time functions for a 25 nm nanoelectrode consumption of 50 mM hexacyanoferrate(III) corresponding to the geometries shown in panels A (blue) and B (green).

**Steady-state Approximation**

The diffusion controlled hexacyanoferrate(III) concentration within the droplets reaches a quasi-steady state. This occurs because the contact radius is small compared to the microdroplet radius, as illustrated in **Figure S5**, which shows the concentration profile at various heights above the contact area for the geometry given in **Figure S4A**. The time it takes to reach this steady-state can be approximated by 12.5*r_0_^9^*, which gives 8 µs for a *r* = 25 nm electrode.


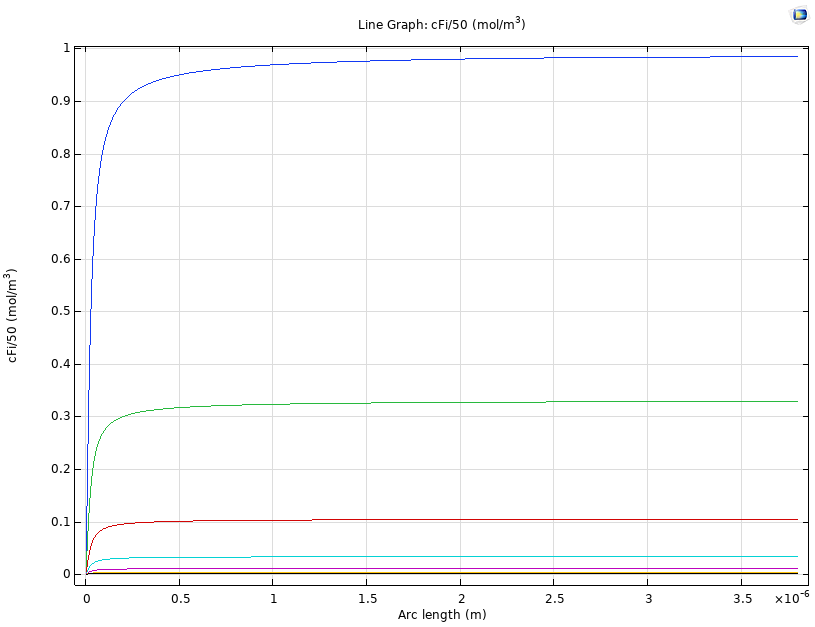


*[C_Fi_]/[C_Fi,0_]*

Height from contact (µm)

**Figure S5.** Overlay of the concentration profiles for the droplet shown in Figure S4A at various times during the consumption: 0.1 s (blue), 0.5 s (green), 1 s (red), 1.5 s (light blue), and 2 s (purple).

**Time-dependent Radius Equation**

Here we describe in detail how we arrive at Equation 4. The previous sections validated that, despite the unique microdroplet geometry, the current is well described by steady-state flux to an inlaid disk. Thus, we begin with the limiting current equation,

$i_{l}=4nFD_{Fi}C_{Fi}r$ *(S4)*

where *i_l_* is the limiting current, *n* is the stoichiometric constant that describes the number of electrons that will transfer per molecule (1 for hexacyanoferrate(III)), *F* is Faraday’s constant, *r* is the radius of the electrode, *D_Fi_* is the diffusion coefficient of hexacyanoferrate(III), and *C_Fi_* is the hexacyanoferrate(III) bulk concentration. The derivation of this equation is well established.^9^

To describe the amperometric (current-time) behavior, we introduce time-dependence in the current term, the bulk concentration term, and the electrode radius term. The electrode radius in our model is the contact radius (*r_c_*).

$i_{l}\left( t \right)=4nFD_{Fi}{r_{c}(t)C}_{Fi}(t)$ *(S5)*

This equation shows that the current, at a given timepoint, is proportional with the (remaining) concentration in the microdroplet and the contact radius at time *t*. If the total volume (*V*) is accessed, the time-dependent concentration term is dictated by the rate of consumption, *i_l_(t),*

$i_{l}\left( t \right)=-nFV\frac{dC_{Fi}}{dt}$ *(S6)*

which can be integrated using the initial condition *C_Fi,0_* (i.e., 50 mM) to arrive at the expression for *C_Fi_(t)*,

$C_{Fi}(t)=C_{0}-\frac{1}{nFV}\int_{0}^{t} i\left( s \right)ds$ *(S7)*

Thus, the concentration at a given timepoint is given by the difference between the initial concentration and the consumed concentration. The consumed concentration is calculated by the integral of the current from the point of initial contact (*t* = 0) to time point *t*. The resulting charge is related to concentration through the *nFV* constants. We can then combine equations S7 and S5 giving,

$i_{l}\left( t \right)=4nFD_{Fi}r_{c}(t){(C}_{Fi,0}-\frac{1}{\mathrm{nFV}}\int_{0}^{t} i\left( s \right)\mathrm{ds})$ *(S8)*

which, when solved for the time-dependent contact radius gives,

$r_{c}\left( t \right)=\frac{i_{l}(t)}{4D_{Fi}nF{(C}_{0}-\frac{1}{nFV}\int_{0}^{t} i\left( s \right)ds)}$ *(S9)*

which is the final form of the equation. This is identical to Equation 4 in the main text, where the more intuitive constant *C_Fi_(t)* replaces ${(C}_{0}-\frac{1}{nFV}\int_{0}^{t} i\left( s \right)ds)$.

Alternatively, one can recognize that *C_Fi_(t)* and ${(C}_{0}-\frac{1}{nFV}\int_{0}^{t} i\left( s \right)ds)$ are also equivalent to $\frac{1}{nFV}\int_{t}^{\infty} i\left( s \right)ds$, giving,

$r_{c}\left( t \right)=\frac{i_{l}(t)}{(\frac{4D_{Fi}}{V}\int_{t}^{\infty} i\left( s \right)ds)}$ *(S10)*

which can be rearranged and presented in terms of microdroplet radius,

$r_{c}\left( t \right)=\frac{\frac{4}{3}\pi R_{drop}^{3}}{4D_{Fi}}\frac{i_{l}\left( t \right)}{\int_{t}^{\infty} i\left( s \right)ds)} \cong$ $r_{c}\left( t \right)=\frac{R_{drop}^{3}}{D_{Fi}}\frac{i_{l}\left( t \right)}{\int_{t}^{\infty} i\left( s \right)ds)}$ *(S11)*

The Eq. S11 form is easily comparable to the equation derived for many examples of pore-widening controlled vesicle release (Eq. S12)^10^.

$R_{pore}\left( t \right)=(\frac{R_{ves}}{\kappa})\left[ (\frac{i_{l}(t)}{\int_{t}^{\infty} i\left( u \right)du}) \right]$ *(S12)*

Where *R_pore_(t)* is the change in the pore radius with time, *R_ves_* is the radius of the vesicle, and constant $\kappa$ describes how quickly a molecule can traverse the diameter of the volume, approximated from the Einstein equation, as shown in equation S13.

$\kappa= \frac{D}{R_{ves}^{2}}$  *(S13)*

By renaming the variables to terms applicable in our system, one can arrive back at equation S11.

# Plotting the Contact Radius as a Function of Time (Full Transients)

**Figure S6** shows the contact radius *versus* time data over full time range of the collision response: From the first current point beyond the baseline to the last current point beyond the baseline. There is a period of stability in the contact radius value that is lost in the final ~0.5 seconds. This significant deviation corresponds to contact radius data that was calculated from experimental current values that are below 3X the noise. Smaller deviations can also be seen for contact radii calculated from current values within 10-3X the noise. Thus, we attribute this observation to error, and do not interpret values of contact radii calculated from current values that were less than 10X the noise.

**B.**

**A.**

**Figure S6.** **(A)** 0 V microdroplet collision analysis: Plot of Equation 4 (right y-axis) where values for *i_lim_* were taken as each sampling point of the experimental data and *Q* was 26.7 fL. The black symbols show the analysis for the entire current transient, the blue symbols show the same analysis but stopped when the values for *i_lim_* fell below 3X the noise (~10 pA), and the red symbols show the same analysis but stopped when the values for *i_lim_* fell below 10X the noise (~35 pA). **(B)** -0.7 V microdroplet collision analysis: Plot of Equation 4 (right y-axis) where values for *i_lim_* were taken as each sampling point of the experimental data and *Q* was 26.7 fL. The black symbols show the analysis for the entire current transient, the blue symbols show the same analysis but stopped when the values for *i_lim_* fell below 3X the noise (~10 pA), and the red symbols show the same analysis but stopped when the values for *i_lim_* fell below 10X the noise (~35 pA).

# Reproducibility of the Dynamic Contact Radius at Highly Negative Overpotentials

**Figures S7** and **S8** are included to demonstrate the reproducibility of the observation: Contact radii forming on a more negatively biased electrode surface (-0.7 V) gradually reach a stable conformation, whereas contact radii forming on a more mildly biased electrode surface (0 V) overshoot the stable conformation and rearrange. Four additional collision responses were analyzed and plotted to complement the data given in the main text. An additional collision response was analyzed for the -0.7 V condition as one trace does not reach a stable contact radius. This unusual response may be due to the influence of the microdroplet surrounding (*e.g.,* interaction with the glass or other microdroplets).


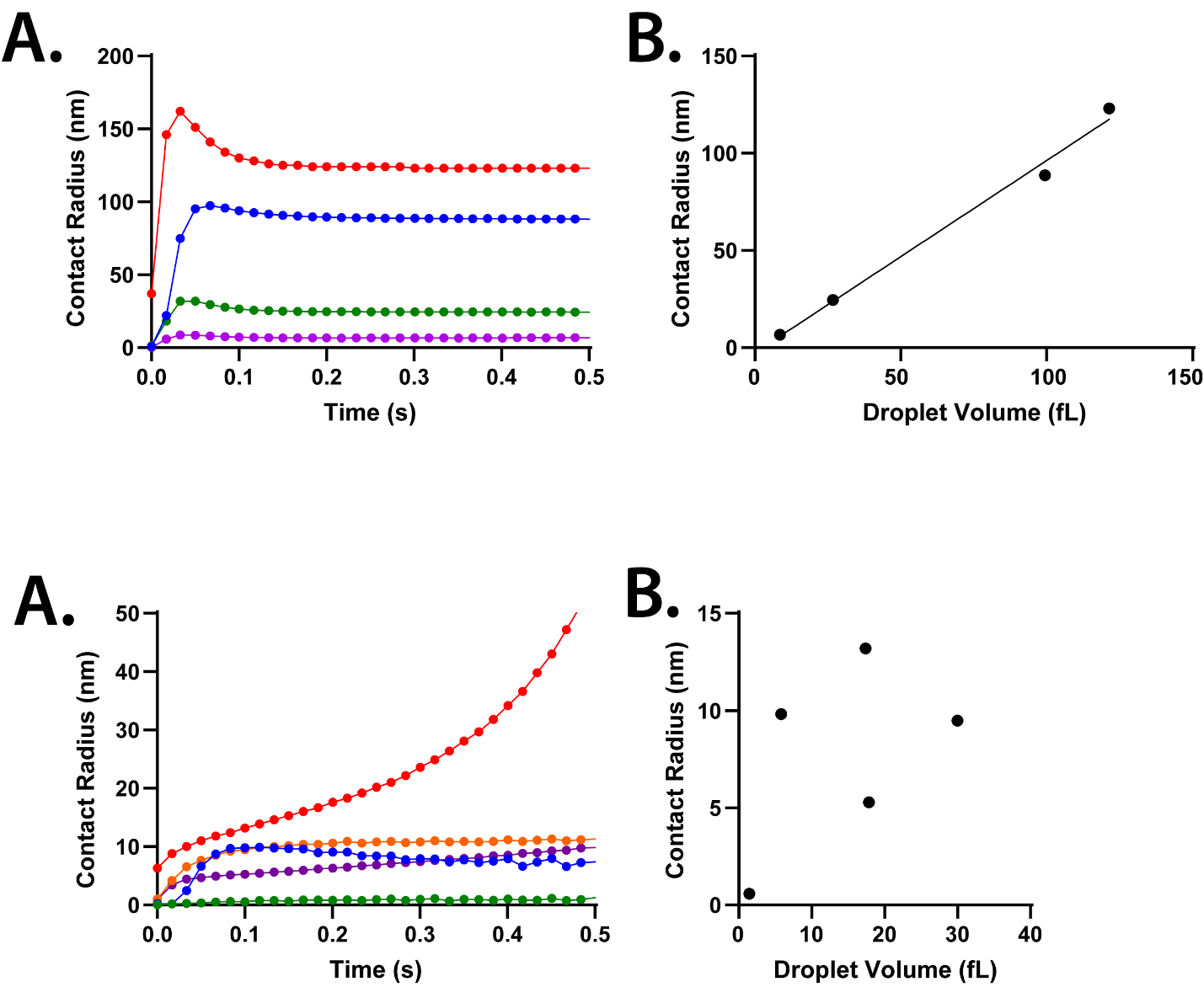


**Figure S7. (A)** Overlay of contact radius versus time for replicate collision events where the platinum microelectrode was biased at -0.7 V. The contact radii were calculated with Equation 4. **(B)** Plot of contact radius at 0.1 seconds versus droplet volume.

# Reproducibility of the Dynamic Contact Radius at Mild Overpotentials


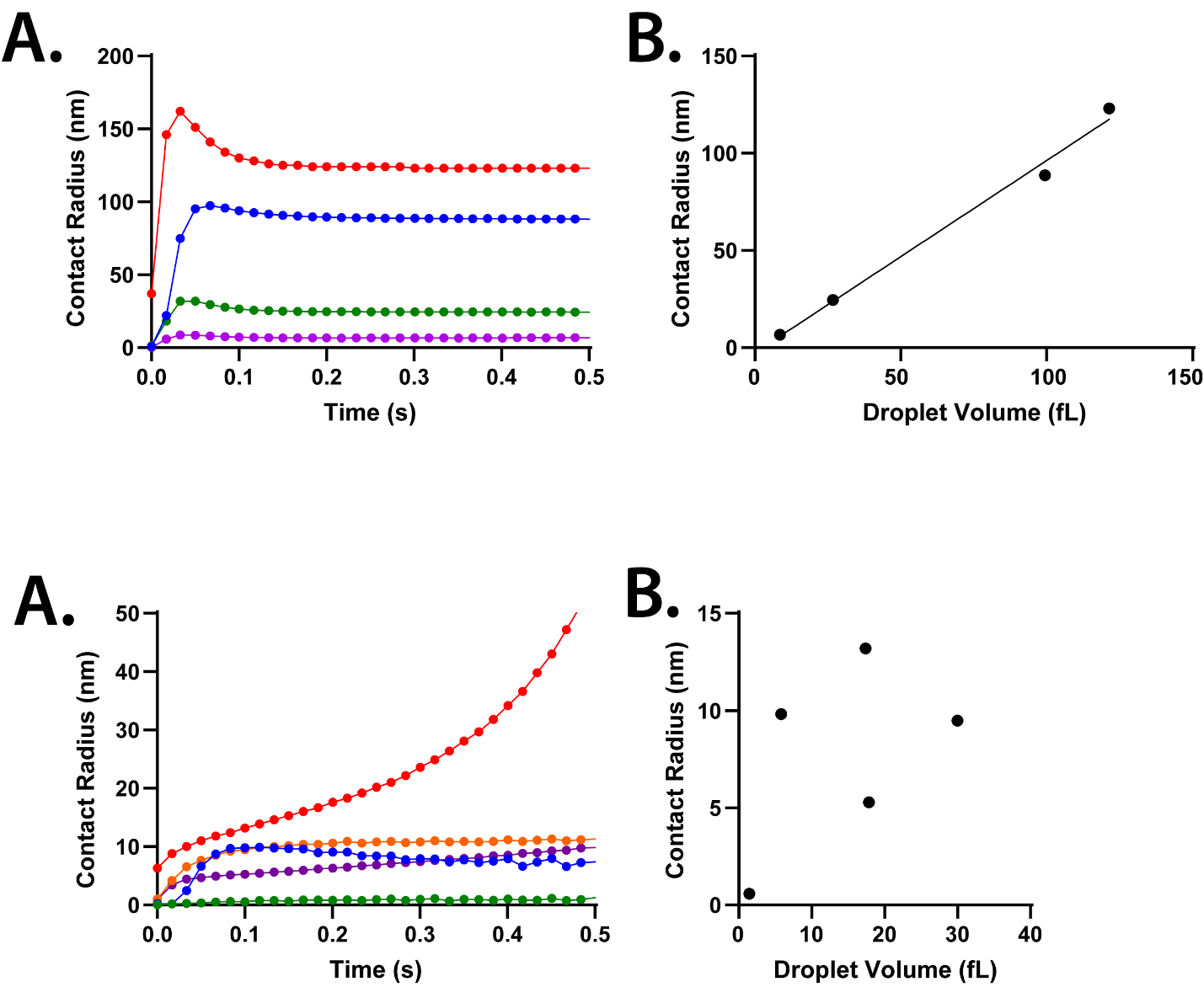


**Figure S8. (A)** Overlay of contact radius *versus* time for replicate collision events where the platinum microelectrode was biased at 0 V. The contact radii were calculated with Equation 4. **(B)** Plot of contact radius at 0.3 seconds *versus* droplet volume. A linear regression is overlaid as a dotted line and the corresponding R^2^ value is 0.99.

# The Dynamic Contact Radius at an Intermediate Overpotential (-0.5 V)

**Figure S9** shows the current *versus* time and calculated contact radius *versus* time plots for collision response collected with a microelectrode that was biased between the two extreme cases presented in the main text (-0.7 V and 0 V). Like the -0.7 V conditions, limiting current from the aqueous phase oxygen reduction is expected to set the baseline current for the collision response for the -0.5 V conditions shown below. A slight overshoot of the contact radius is observed; a shape with intermediate characteristics of the typical 0 V and -0.7 observations.


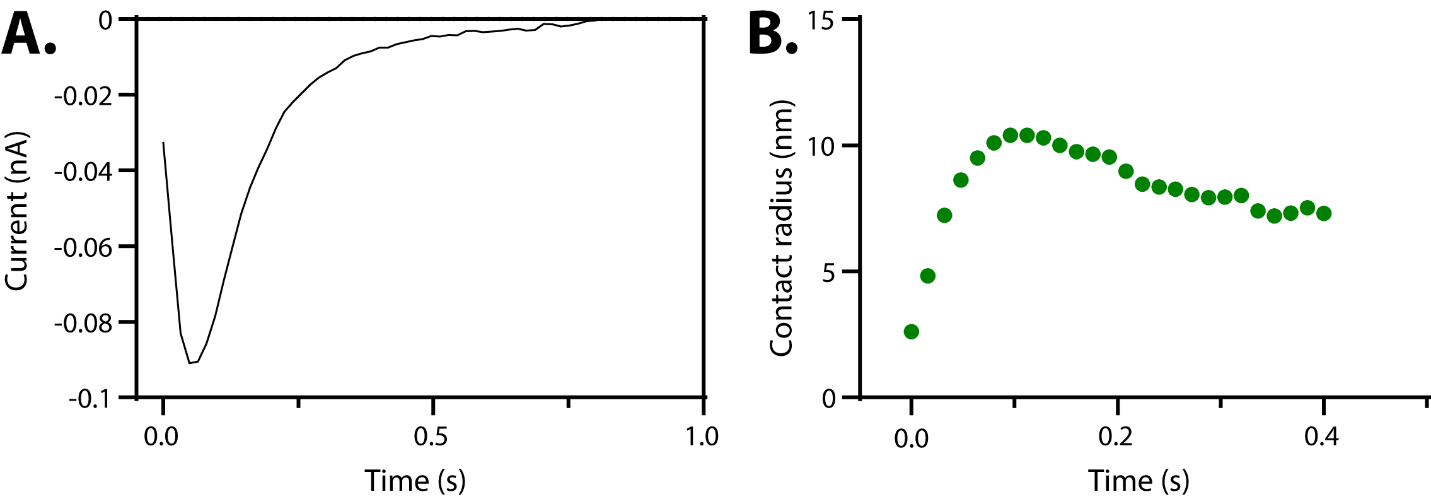


**Figure S9. (A)** Example background-subtracted transient collected with the working electrode biased at -0.538 V *versus* sat’d Ag/AgCl (adjusted to the 1 M KCl potential scale). The first point shown is the first point beyond the baseline. A three-electrode setup was used with a platinum microelectrode (10 µm diameter) as the working electrode, a leakless sat’d Ag/AgCl reference electrode and a platinum wire counter electrode. The emulsion solution consists of 50 mM hexacyanoferrate(III) and 100 mM sodium perchlorate in 0.1 M tetrabutylammonium perchlorate in dichloroethane. The sampling rate is 60 Hz. **(B)** Plot of contact radius *versus* time for replicate collision events where the platinum microelectrode was biased at -0.538 V. The contact radii were calculated with Equation 4.

# Overpotential-dependent Collision Response of Microdroplets Under Anaerobic Conditions

We show a clear qualitative trend for the voltage dependence of microdroplet wetting, in the absence of any background reactions. The experiments represented in **Figure 1** were performed in an Argon-purged glovebox and the representative collision events indicated in **Figure 1*i-iii*** were highlighted as they each reached a similar stable contact radius (7 nm). The wetting dynamics leading to this stable contact are shown in the Equation 4 analyses given in **Figure S10**. Importantly, the same trend in the potential dependency is observed in anaerobic conditions as in ambient conditions, suggesting that the mechanism causing the current decay shape (we propose microdroplet wetting) is not significantly impacted by the current contributions from the oxygen reduction reaction.


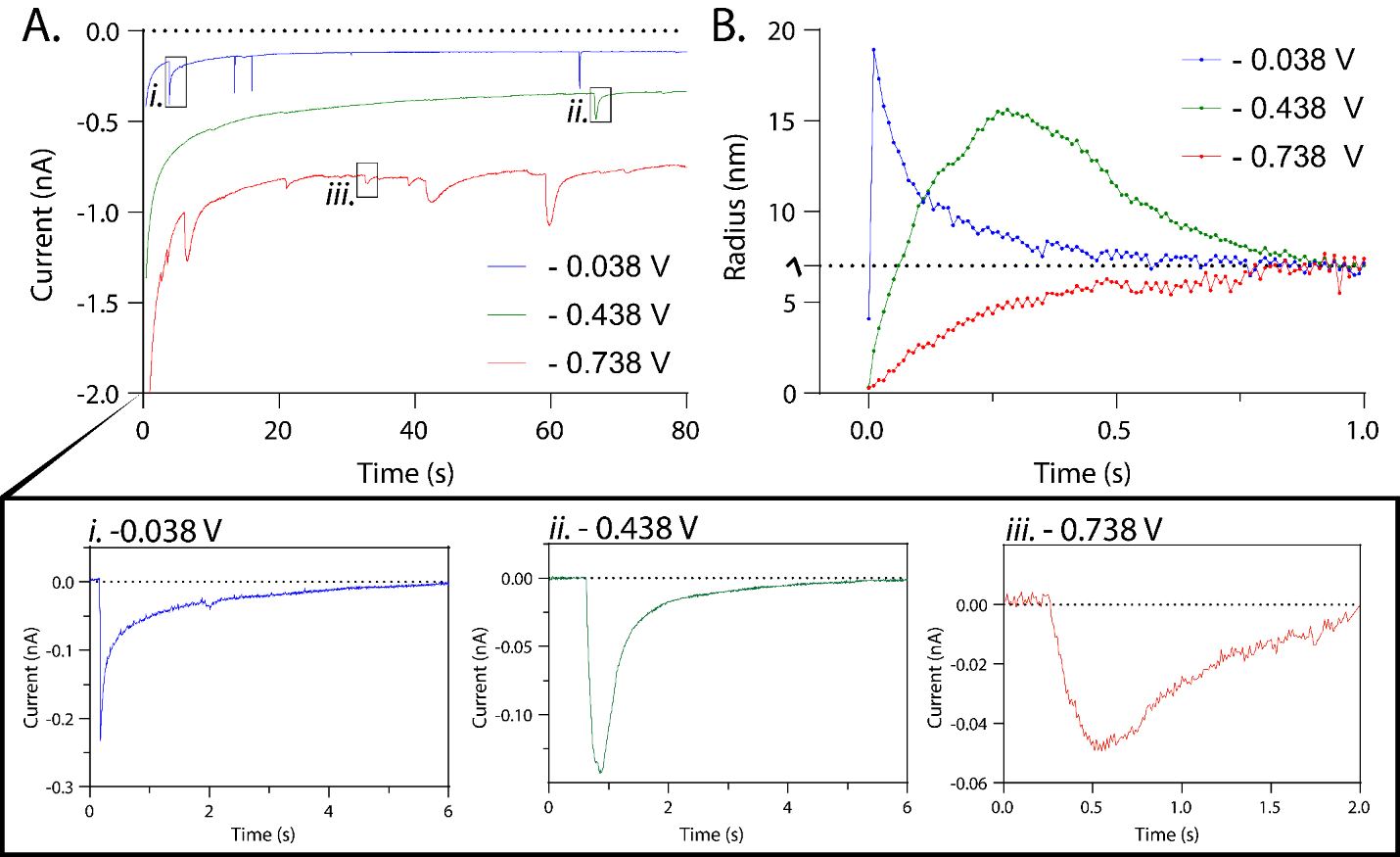


**Figure S10.** Plot of Equation 4 for collision responses collected in an Argon-purged glovebox. Data from collision responses collected with a microelectrode bias of -0.04 V (blue), -0.44 V (green) and -0.74 V (red) versus Ag/AgCl are overlaid for comparison.

# Overpotential-dependent Collision Response of Nanodroplets

It is more difficult to draw quantitative conclusions about the wetting dynamics of nanodroplets (droplets with radii smaller than 1 micron), as these collisions pass smaller currents. **Figure S11** qualitatively shows that the shape of the transients for the nanodroplets follows the same trend as the microdroplets: At low overpotentials the peak is sharp, and the decay looks exponential. At more negative overpotentials, the transient becomes rounded, and the exponential decay appears after the first ~100-200 ms. While it is plausible that the size of the colliding droplet could influence the wetting dynamics, we did not find clear evidence of this relationship in this study.


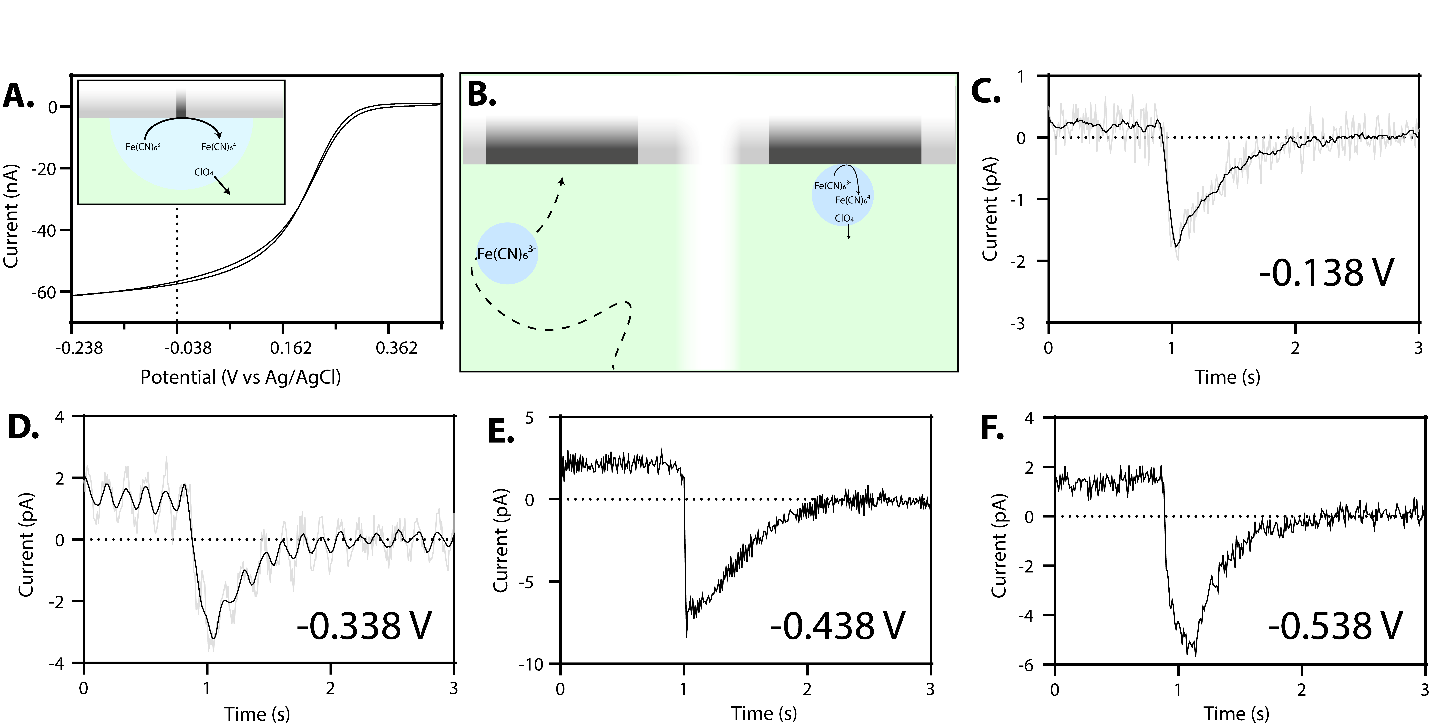


**Figure S11.** Representative collision responses of nanodroplets under ambient conditions. For all electrochemical data in this figure, the working electrode was 5 µm platinum microelectrode, the reference electrode was a leakless Ag/AgCl (sat’d KCl adjusted to the 1 M KCl scale), and the counter electrode was a platinum wire. **(A)** Cyclic voltammogram collected in a 5 µL aqueous droplet of 50 mM potassium hexacyanoferrate and 100 mM sodium perchlorate adsorbed to a platinum microelectrode and submerged in a DCE solution containing 500 mM tetrabutylammonium perchlorate. The scan rate was 50 mV/s. **(B)** Schematic showing the collision experiment where nanodroplets diffuse to the microelectrode surface and irreversible adsorb and hexacyanoferrate is reduced. Perchlorate is suggested to transfer out of the droplet to maintain charge neutrality. **(C-D)** Background subtracted representative collision responses collected at various overpotentials. Smoothing was applied in panels C and D where the unsmoothed data is included in grey. The sample rate was 100 Hz.

# Reducing the Electrostatic Repulsion by Charging the Microdroplet

We suggest that electrostatic repulsion can be mitigated by either biasing the electrode less negatively (as is done throughout this work) or by decreasing the negative charge on the microdroplets. It is well established that acidification of the aqueous phase in an aqueous|oil emulsion increases the microdroplet surface charge.^11^ Thus, we added 0.2 mM of perchloric acid into the 50 mM hexacyanoferrate(III), 100 mM sodium perchlorate aqueous phase. More acidic microdroplets were not investigated due to the instability of hexacyanoferrate at low pH, which can liberate (dangerous) HCN over time. These experiments were performed in a glovebox after the bulk phases sat under Argon atmosphere overnight. **Figure S12** illustrates the collision response at -0.74 V, which follows a sharp decay function. The sharp decay of the acidic microdroplet collisions clearly contrast with the rounded transients that are observed for collisions under the same potential conditions but in neutral pH (**Figure S13**). In fact, they more closely resemble the collision response of the neutral microdroplets on the more mildly biased electrode. This in line with the hypothesis that reducing the electrostatic repulsion between the microelectrode and microdroplet surfaces changes the wetting profile.


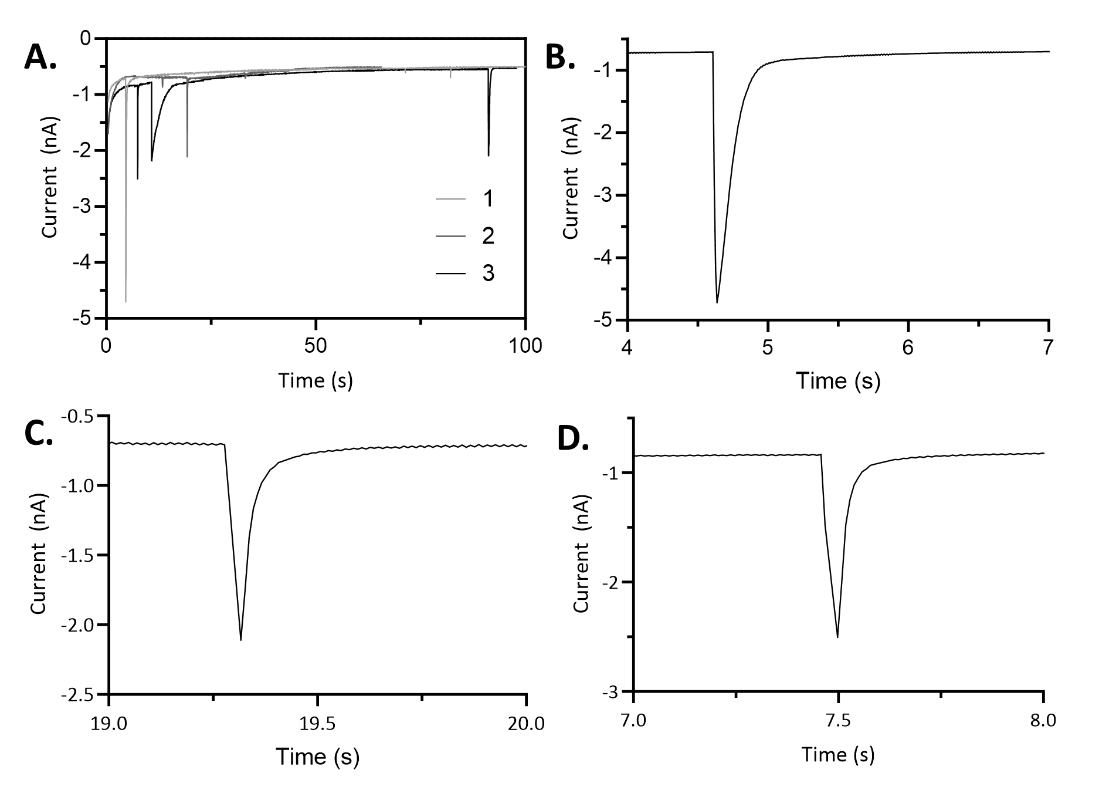


**Figure S12.** Overlay of replicate amperograms collected with the working electrode biased at -0.738 V versus Ag/AgCl. **(B-D)** Example transients from each amperogram. For all panels in this figure, a three-electrode setup was used with a platinum microelectrode (25 µm diameter) as the working electrode, a leakless Ag/AgCl reference electrode (sat’d KCl converted to 1 M KCl potentials) and a platinum wire counter electrode. The emulsion solution consists of 80 µL 50 mM hexacyanoferrate(III), 0.2 mM perchloric acid, and 100 mM sodium perchlorate in 5 mL 0.5 M tetrabutylammonium perchlorate in dichloroethane. The sampling rate is 100 Hz.


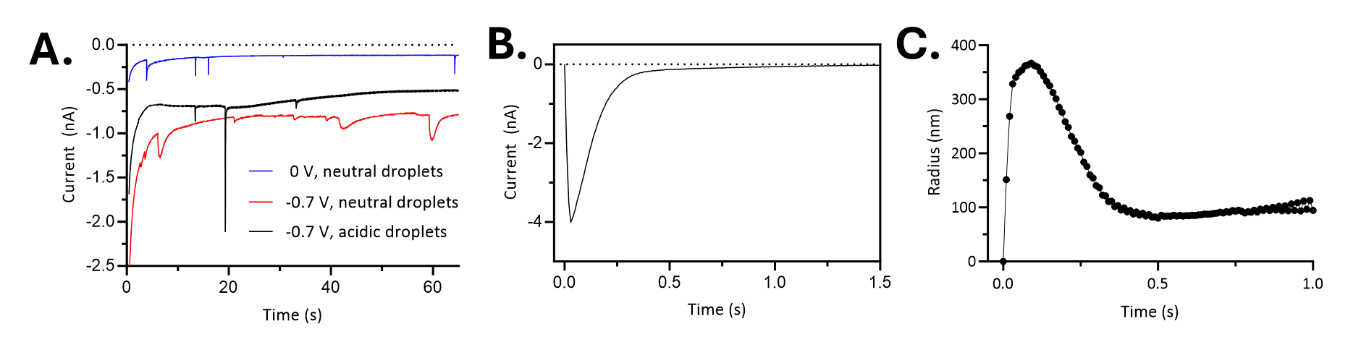


**Figure S13. (A)** Overlay of representative amperograms collected in an Argon saturated glovebox. The working electrode was biased at -0.738 V versus Ag/AgCl to collect collisions of neutral microdroplets (red,reproduced from main text Figure 1) and acidified microdroplets (trace 2 in Figure S12). The working electrode was biased at -0.038 V versus Ag/AgCl to collect collisions of neutral microdroplets (blue, reproduced from main text Figure 1). For all panels in this figure, a three-electrode setup was used with a platinum microelectrode (25 µm diameter) as the working electrode, a Ag/AgCl reference electrode (sat’d KCl converted to 1 M KCl potentials) and a platinum wire counter electrode. The sampling rate is 100 Hz. **(B)** Background subtracted transient from the black trace in Panel A. **(C)** Plot of Equation 4 for the collision response in Panel B.

**A.**

# Current Transients Measured in a Glovebox

It is our assumption that the limiting current observed after the blip-type consumption event in the amperogram arises from the partitioning of oxygen from the dichloroethane continuous phase (exposed to normal atmospheric conditions) into the microdroplet, and reduction of the aqueous-phase oxygen at the contact area. To confirm this, we made the emulsion and performed the collision experiment in an argon-purged glovebox during a collision experiment. The glovebox atmosphere was kept below 5 ppm of oxygen during all experiments, though some residual oxygen may be present in the organic solvent (**Figure S12**). In line with our expectations, we find that that collisions under limited oxygen conditions return to the original baseline (**Figure S13A**). We also find that the shape of the transients exhibits the same bias-dependent behaviour that we attribute to wetting (**Figure S13B)** regardless of the oxygen atmosphere, suggesting the oxygen reduction reaction can be treated as a background process in the ambient experiments.


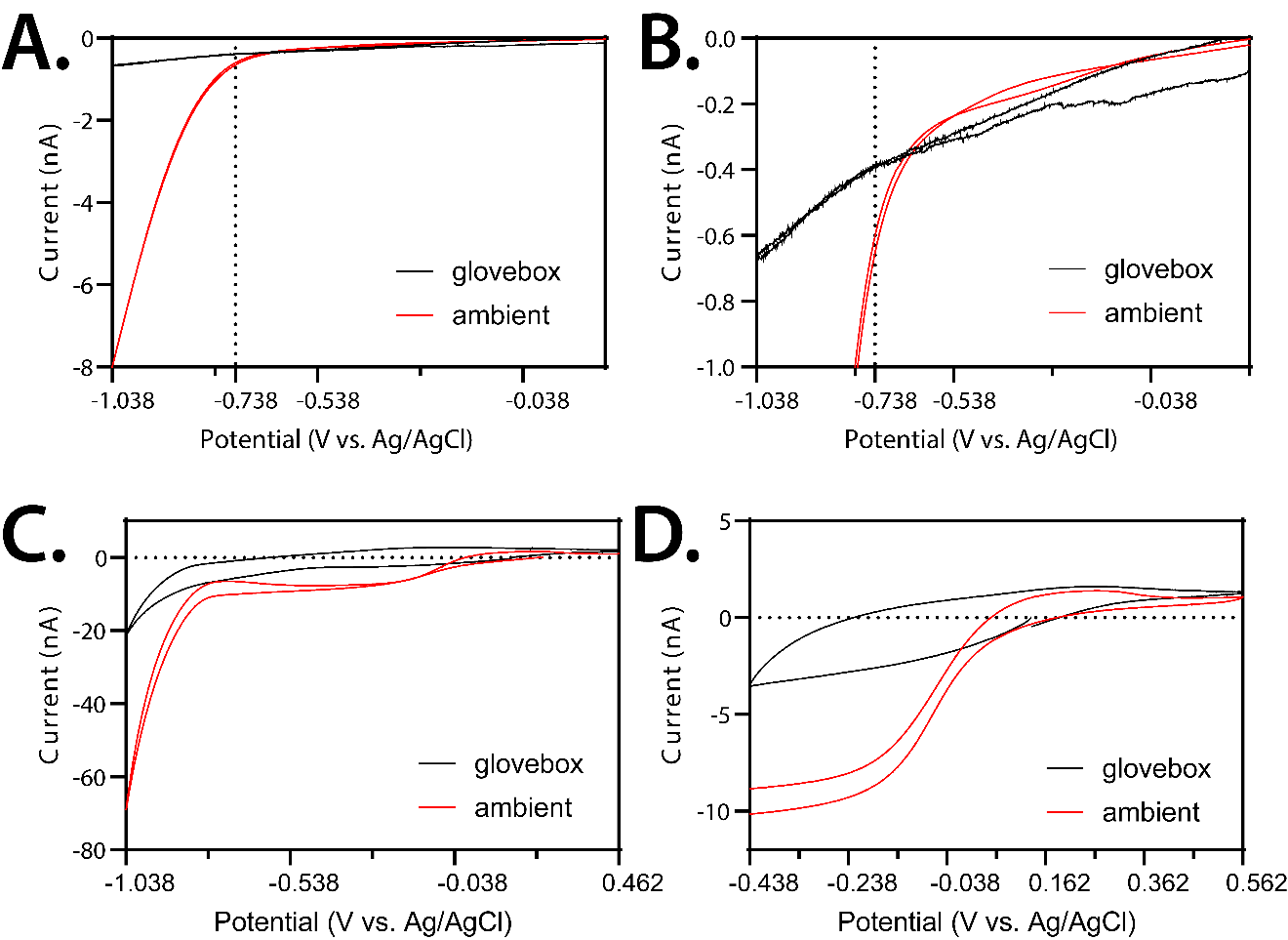


**Figure S14.** **(A)** Overlay of cyclic voltammetry in 0.5 M TBAP DCE under ambient conditions (benchtop, red) and in an argon purged glovebox (black). **(B)** Zoom-in on Panel A to show the current at relevant potentials. **(C)** Cyclic voltammograms of 0.1 M NaClO_4_ under ambient conditions (benchtop, red) and in an argon purged glovebox (black). **(D)** Cyclic voltammetry of 0.1 M NaClO_4_ performed in a smaller window compared to Panel C. For all panels in this figure a three-electrode setup was used with a platinum microelectrode (*d* = 25 µm) working electrode, a platinum wire counter electrode and a leakless Ag/AgCl reference electrode (sat’d KCl adjusted to the 1 M KCl potential scale). The scan rate was 20 mV/s.

**
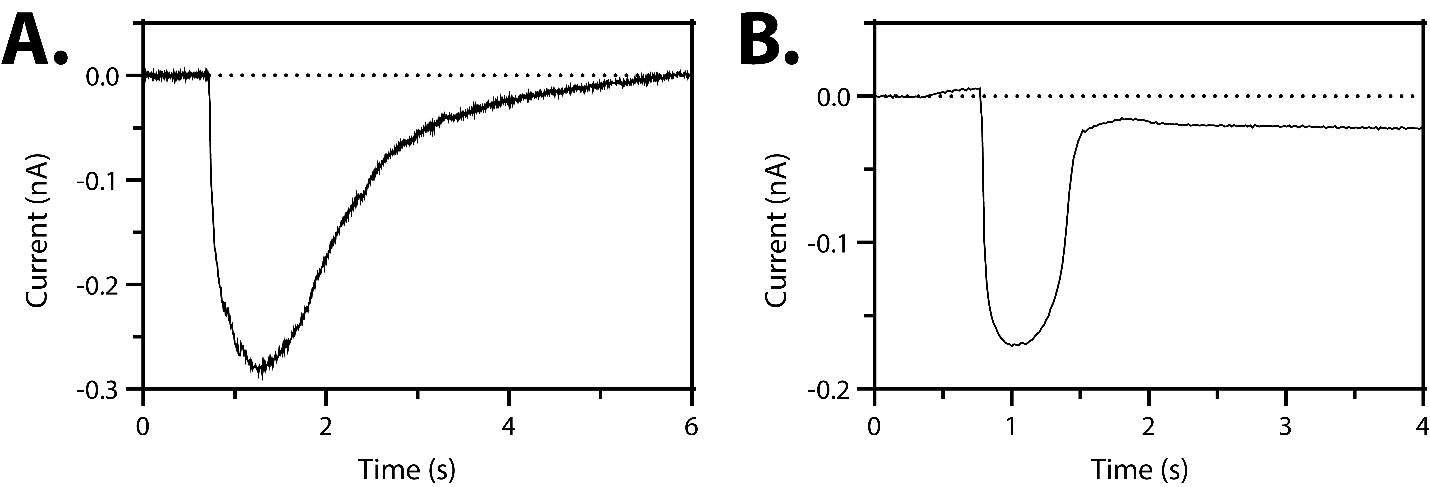
**

**Figure S15.** **(A)** Background-subtracted transient collected in a glovebox (O_2_ < 5 ppm). The collision was collected with a platinum microelectrode (*d* = 25 µm) biased at -0.738 V, a leakless Ag/AgCl reference electrode, and a platinum wire. The sample rate was 10 ms. **(B)** Background-subtracted transient collected in ambient air. The collision was collected with a platinum tp microelectrode (*d* = 10 µm) biased at -0.7 V, a Ag/AgCl reference electrode, and a platinum wire. The sample rate was 60 Hz.

# Analyzing the Current from the Reduction of Dissolved Oxygen in the Microdroplet

As shown in **Figure S14**, oxygen reduction in the aqueous phase occurs at mass transfer limited rates beyond ~ -0.4 versus Ag/AgCl. However, only the current involved in consumption is used in calculating the microdroplet size and the contact radius. Because the oxygen can partition from dichloroethane into the aqueous microdroplet, there is no consumption of the dissolved oxygen. Thus, the current contribution from oxygen reduction reaction is stepwise, resulting in a new limiting current observed after the current blip from the hexacyanoferrate(III) consumption (**Figure S16**, red line). This new limiting current is used as the baseline, and is subtracted from each current value to give the contribution of only hexacyanoferrate(III) reduction.

**
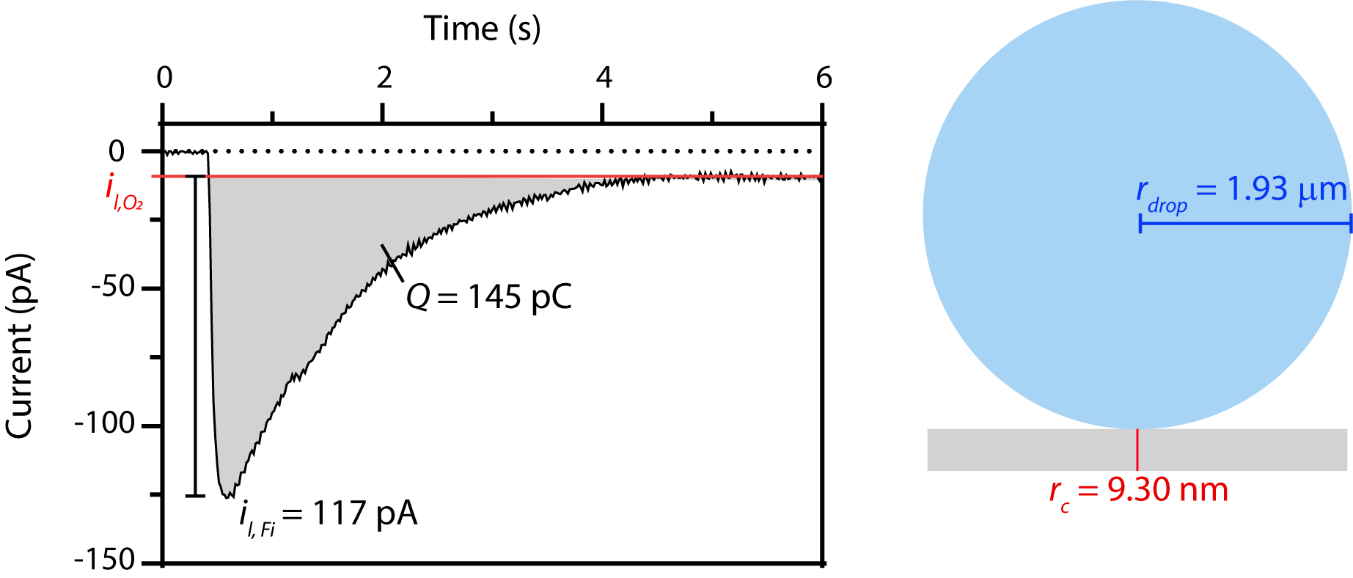
**

**Figure S16**. (**A**) Example background subtracted transient with overlaid measurements. The limiting current from oxygen reduction (*i_l,O2_*) is labelled as a red line at x = -9.45 pA. This acts as the baseline for the determination of the limiting current due to hexacyanoferrate reduction (*i_l,Fi_*). The grey shading illustrates the integration giving the charge passed (*Q*). (**B**) Scheme of microdroplet dimensions as determined from the measurements in panel A. The microdroplet radius (*r_drop_*) is determined to be 1.93 µm and the contact radius (*r_c_*) is determined to be 9.30 nm.

# Estimating the Dissolved Oxygen Content in Individual Microdroplets

If one assumes that the oxygen reduction reaction at the microdroplet contact is limited by diffusion, and not electron transfer or partitioning kinetics, one can use the stepwise increase in the limiting current before and after the current transient to calculate the concentration of dissolved oxygen in the microdroplet (**Figure S17A**).Because the reaction mechanism in such a multiphase environment (Dichloroethane|aqueous|platinum) is difficult to know with certainty, the table in **Figure S17B** uses a range of *n* values, which correspond to various possible mechanisms of electron transfer.

**A.**

**B.**

**
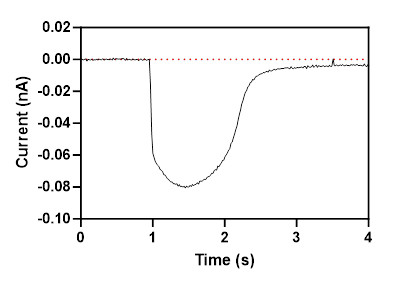
**

**Figure S17.** **(A)** Representative current transient collected with a Pt tip microelectrode collected with a three-electrode setup with a platinum working electrode (10 µm diameter) based at -0.7 V versus a Ag/AgCl reference electrode, and a platinum wire counter electrode. In line with IUPAC convention, cathodic current is represented as negative. **(B)** Table calculating the contact radius (r_c_) from i_l(Fi)_, the concentration of dioxygen C_O2_ from the calculated contact radius for six different transients assuming n = 1 and n =4.

# References

(1) Colón-Quintana, G. S.; Clarke, T. B.; Dick, J. E. Interfacial solute flux promotes emulsification at the water|oil interface. *Nature Communications* **2023**, *14* (1), 705. DOI: 10.1038/s41467-023-35964-9.

(2) Markin, V. S.; Volkov, A. G. The gibbs free energy of ion transfer between two immiscible liquids. *Electrochimica Acta* **1989**, *34* (2), 93-107. DOI: <https://doi.org/10.1016/0013-4686(89)87072-0>.

(3) Moazzenzade, T.; Yang, X.; Walterbos, L.; Huskens, J.; Renault, C.; Lemay, S. G. Self-Induced Convection at Microelectrodes via Electroosmosis and Its Influence on Impact Electrochemistry. *Journal of the American Chemical Society* **2020**, *142* (42), 17908-17912. DOI: 10.1021/jacs.0c08450.

(4) Terry Weatherly, C. K.; Glasscott, M. W.; Dick, J. E. Voltammetric Analysis of Redox Reactions and Ion Transfer in Water Microdroplets. *Langmuir* **2020**, *36* (28), 8231-8239. DOI: 10.1021/acs.langmuir.0c01332.

(5) Li, Y.; Deng, H.; Dick, J. E.; Bard, A. J. Analyzing Benzene and Cyclohexane Emulsion Droplet Collisions on Ultramicroelectrodes. *Analytical Chemistry* **2015**, *87* (21), 11013-11021. DOI: 10.1021/acs.analchem.5b02968.

(6) Kim, B.-K.; Kim, J.; Bard, A. J. Electrochemistry of a Single Attoliter Emulsion Droplet in Collisions. *Journal of the American Chemical Society* **2015**, *137* (6), 2343-2349. DOI: 10.1021/ja512065n.

(7) Sabaragamuwe, S. G.; Madawala, H.; Puri, S. R.; Kim, J. Towards ultralow detection limits of aromatic toxicants in water using pluronic nanoemulsions and single-entity electrochemistry. *Analytica Chimica Acta* **2020**, *1139*, 129-137. DOI: <https://doi.org/10.1016/j.aca.2020.09.053>.

(8) Einstein, A. On the motion of small particles suspended in liquids at rest required by the molecular-kinetic theory of heat. *Annalen der physik* **1905**, *17* (549-560), 208.

(9) Bard, A. J.; Faulkner, L. R.; White, H. S. *Electrochemical methods: fundamentals and applications*; John Wiley & Sons, 2022.

(10) Oleinick, A.; Svir, I.; Amatore, C. 'Full fusion' is not ineluctable during vesicular exocytosis of neurotransmitters by endocrine cells. *Proc Math Phys Eng Sci* **2017**, *473* (2197), 20160684. DOI: 10.1098/rspa.2016.0684 From NLM.

(11) Wu, Y.; Li, Q.; Deng, F.; Liang, X.; Liu, H. Solvent Effect on ζ Potential at an Aqueous/Oil Interface in Surfactant-Free Emulsion. *Langmuir* **2014**, *30* (8), 1926-1931. DOI: 10.1021/la403900e.
